# Have the media influenced the use of hip resurfacing arthroplasty? A review of UK print media

**DOI:** 10.1308/003588412X13171221592014

**Published:** 2012-09

**Authors:** A Malviya, GH Stafford, RJF Villar, RN Villar

**Affiliations:** ^1^Spire Cambridge Lea Hospital,UK; ^2^Reuters Television, Tokyo,Japan

**Keywords:** Printed media, Hip replacement arthroplasty, Trends

## Abstract

**INTRODUCTION:**

The aim of this study was to look at the different claims made about hip resurfacing arthroplasty in the popular UK print media and how this relates to findings in the scientific literature.

**METHODS:**

A review of UK popular print media from January 1992 to June 2011 was performed using the Lexis® Library online news database. Only articles discussing the clinical results of hip resurfacing arthroplasty were included. After excluding duplicates, 49 newspaper articles were found suitable for this study. The main outcome measure was the claims made in popular UK print media about hip resurfacing. These were compared with the scientific publication. We reviewed the trend of use of hip resurfacing prostheses during the same period as reported in the National Joint Registry.

**RESULTS:**

A disparity was found between the claims in the newspapers and published scientific literature. The initial newspaper articles highlighted only the positive aspects of hip resurfacing arthroplasty, without definitive contemporary evidence backing the claims. Most of these claims were refuted by future scientific publications. The initial positive media reports coincided with an increase in the use of hip resurfacing but the decline coincided with negative reports in the scientific literature.

**CONCLUSIONS:**

The trend of the newspaper articles and that of the number of hip resurfacing prostheses implanted suggests that the media may have been partly responsible for the increased use of this prosthesis. The subsequent decrease was initiated by the scientific literature.

The media can influence public perception of medical treatment. In plastic surgery popular media have been shown to alter the attitude and decision making of patients.^1,2^ Although the media play a powerful role in affecting patients' opinions and feelings, the physician–patient communication and the decision making process have been shown to be related primarily to the level of education.^3^ Concern has been raised that media reports may have substantial errors.^4^ The news media have been shown to significantly misrepresent scientific findings and this may contribute to the public’s distorted perceptions of health hreats.^5^ Modern-day patients are more informed about the various options available. This information may occasionally drive the growth of certain forms of treatment offered to the patient.

We wished to explore the influence of the media on the choice of prosthesis in orthopaedic surgery. We selected hip resurfacing as the index procedure because the prosthesis is a relatively new device that over the last 20 years has received much media attention as well as raising debate in the scientific literature. The aim of this study was to look at the different claims made about hip resurfacing arthroplasty in the popular UK print media and how this relates to findings in the scientific literature.

## Methods

The search for newspaper articles on hip resurfacing arthroplasty was performed by a qualified journalist (RJFV) using the Lexis® Library online news database. The content of these articles was further checked for their relevance and various claims were explored. The articles were subjectively assessed and grouped into those that highlighted the potential benefits (positive publicity) of hip resurfacing arthroplasty and those that discussed the problems (negative publicity). Details of the number of hip resurfacing arthroplasties performed in recent years were obtained from the National Joint Registry of England and Wales (NJR).^6^ The different claims made by the newspapers since the introduction of resurfacing were matched with the results that subsequently appeared in the scientific literature.

The Lexis® Library online news database stores articles published in the UK national newspapers. The papers included with the range of dates stored in the database were:

> *Daily Mail* and *The Mail on Sunday* (1 January 1992 – June 2011)
> *Daily Star* and *Daily Star* *Sunday *(15 December 2000 – June 2011)
> *The Daily Telegraph* and *The Sunday Telegraph* (30 October 2000 – June 2011)
> *The Guardian* (14 July 1984 – June 2011)
> *The Observer* (2 January 1995 – June 2011)
> *The Independent* and *Independent on Sunday* (19 September 1988 – June 2011)
> *The Times* and *The Sunday Times* (1 July 1985 – June 2011)
> *Daily Mirror* and *Sunday Mirror* (29 May 1985 – June 2011)
> *The Sun* (1 January 2000 – June 2011)

Search 1 with major mentions (in headline, opening line or first paragraph) of ‘hip’ AND major mentions of ‘replacement’ AND mentions (anywhere in text) of ‘technique’ revealed 71 articles, search 2 with three or more mentions of ‘hip replacement’ in the text revealed 38 articles, search 3 with ‘BHR’ AND ‘hip’ anywhere in the body of the text revealed 8 articles, search 4 with ‘Birmingham hip’ anywhere in the body of the text revealed 55 articles and search 5 with ‘hip resurfacing’ anywhere in the body of the text revealed 111 articles.

Only articles discussing the clinical results of hip resurfacing arthroplasty were included in the study and those that were generic for hip replacements or that reported the financial performance of the companies in the stock market were excluded. After excluding duplicates, 49 newspaper articles were found suitable for this study. Two observers (AM and GHS) were involved in the shortlisting process to reduce the risk of bias.

The circulation figures for the newspapers were obtained from the Audit Bureau of Circulations (ABC).^7^ The figure obtained for the day of any one newspaper report is a mean daily circulation for the month. We therefore had two figures: one for the relevant newspaper (in which the article was published) and the other for the total number of UK newspapers published and circulated on that day. These were used to calculate figures for the percentage of newspapers carrying the relevant article.

## Results

Of the 49 newspaper articles published from 1996 to 2011, the first 35 (1996 to April 2008) had positive publicity about hip resurfacing. From 2004 to 2008 concerns regarding hip resurfacing arthroplasty and the potential consequences of metal ion issues started appearing in the scientific literature.^8–13^ Boardman *et al* reported the first case of a psoas mass associated with hip resurfacing arthroplasty in 2006.^8^

During the same year, Hart *et al* demonstrated the association between metal ions in hip resurfacing arthroplasty and a reduced T-cell count.^9^ The negative reports were gradually reflected in the popular press and after August 2008 there were 14 more newspaper reports on hip resurfacing arthroplasty, 12 of which highlighted the negative aspects of the implant.

Twelve of the articles were published in the Sunday newspapers. These have a mean circulation of 1.34 million and represent 11.1% of the total number of newspapers circulated on that day. Thirty-seven of the articles were published on weekdays. These have a mean circulation of 1.33 million and represent 11.4% of the total number of newspapers circulated on that day.

**Figure 1:**
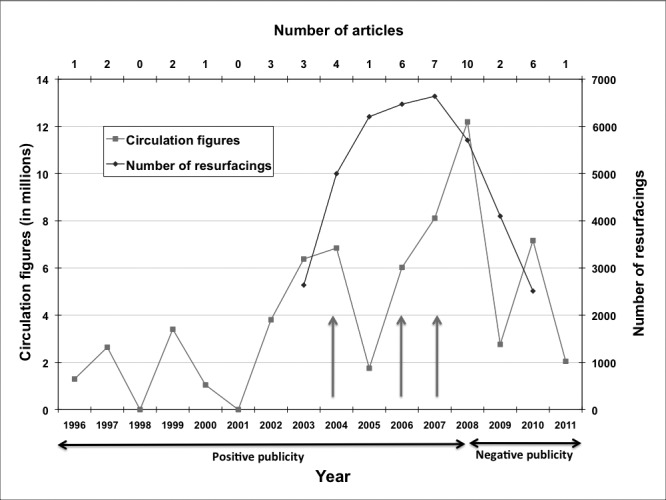
Annual circulation figures for the newspapers, annual number of articles and number of hip resurfacing arthroplasties performed. The red arrows represent the following published papers raising concerns about metal-on-metal hip resurfacing: 2004 – MacDonald;^13^ 2006 – Boardman *et al*,^8^ Hart *et al*;^9^ 2007 – Lachiewicz,^10^ Ziaee *et al*. ENREF 9^11^

Figure 1 depicts the annual circulation figures for all the articles published in that year with the number of articles and the number of hip resurfacing arthroplasties performed in the UK from 2003 to 2009. From 2003 to 2007 there was an increase in the number of hip resurfacing arthroplasties from 2,638 to 6,638 per year. This figure declined to 5,707 (8% of all hip replacements) in 2008 and 4,099 (6% of all hip replacements) in 2009.^6^

The following are the claims made by the newspaper articles and the current evidence.

### ‘Everlasting hip’

In a 1996 article entitled *Everlasting hip removes pain of replacements*, *The Sunday Times* stated that the recoating technique with metal would not lead to wear and would last forever.^14^ This was repeated in other publications^15,16^ followed by subsequent reports that hip resurfacing would last longer than conventional total hip replacement.^17–19^

### Current evidence

A systematic review of hip resurfacings from 2011 found that none of the hip resurfacing arthroplasty implants used met the full 10-year National Institute for Health and Clinical Excellence (NICE) benchmark of survival and only some studies (13 of 29) showed satisfactory survival against the 3-year NICE benchmark.^20^ In a technology overview of metal-on-metal hip resurfacing arthroplasty, which looked at the results of the Australian Orthopaedic Association National Joint Replacement Registry, the Swedish Hip Arthroplasty Register and the NJR, it was concluded that the revision rate for hip resurfacing arthroplasty was higher at three and seven years compared with conventional hip replacements.^21^ The outcome for revision after revision for metal wear related failure of metal-on-metal hip resurfacing arthroplasty is poor.^22^

### ‘For younger, active patients’

In 1997 *The Observer* stated: ‘… hip replacement operations could soon be unnecessary for tens of thousands of younger people crippled by arthritis. Relining damaged joints achieves the same effects as total replacement, but is far less invasive – and far more liberating.’^23^ Similar claims were made in other articles^18,19,24–30^ with one article stating that impact may prolong the hip’s life and improve the bone density.^31^

### Current evidence

A systematic review comparing metal-on-metal resurfacing arthroplasty with standard total hip replacement revealed that the hip functional outcome scores were similar for both groups but that the activity level of the resurfacing arthroplasty group was higher.^32^ However, the results indicated increased rates of revision, femoral neck fractures and component loosening among patients who received a modern metal-on-metal hip resurfacing arthroplasty. The authors concluded that there was insufficient evidence to determine whether modern metal-on-metal total hip resurfacing arthroplasty offers clinical advantages over standard total hip replacement.

### ‘Less invasive surgery’

In 1997 in ‘Don't get a new hip, just reline the old one’, the *Daily Mail* claimed that hip resurfacing arthroplasty is a simpler and less invasive operation.^30^ The same claim was also made by *The Independent* in March 2006.^33^

### Current evidence

In a study comparing hip resurfacing arthroplasty with hip replacement, Vendittoli *et al* found that the mean incision length for hip resurfacing arthroplasty patients was 17.2cm compared with 14.5cm for hip replacement patients although the difference was not statistically significant (*p*=0.382).^34^

### ‘Less bone resected’

In October 1997 *The Observer* claimed that the amount of bone resected in resurfacing arthroplasty has been estimated to be the size of a knuckle compared with a fist for total hip replacement.^23^ A similar claim was made in the same month by the *Daily Mail*.^30^

### Current evidence

A cadaver study showed that the amount of acetabular bone loss during resurfacing arthroplasty was the same as for a conventional total hip replacement but that the resurfacing arthroplasty component resulted in approximately three times less bone removal from the femur.^35^ Loughead *et al* reported that more acetabular bone is removed during hip resurfacing arthroplasty than during hybrid hip replacement, especially in patients with a larger femoral head.^36^ In contrast, Moonot *et al* did not find any difference in acetabular component size for patients undergoing hip resurfacing arthroplasty compared with those having an uncemented hip replacement.^37^

### ‘Lower risk of dislocation’

In July 2003 the *Daily Mirror* claimed that hip resurfacing arthroplasty had practically no problems with dislocation.^38^ Other articles stated that the procedure has a low dislocation rate,^17,27^ with one quoting a figure of 0.05%.^24^

### Current evidence

In a multi-surgeon series of 5,000 hip resurfacing arthroplasties, it was found that the prevalence of dislocation was 0.1%, with it being significantly more common in women (*p*=0.004).^39^ In a systematic review comparing hip resurfacing arthroplasty with hip replacements in young active patients, the incidence of dislocation was higher at one and two years for total hip replacements but this was not statistically different.^32^

### ‘Less time in surgery’^17^

In August 2002 *The Times* claimed that the resurfacing arthroplasty procedure takes half the time to perform.^24^

### Current evidence

Studies comparing hip resurfacing arthroplasty with total hip replacements have found that the surgical time was significantly longer for hip resurfacing (*p*<0.001)^34^ and on average 18% longer than for total hip replacements.^40^

### ‘Fewer post-operative thromboses’^17^

In August 2002 *The Times* stated that hip resurfacing arthroplasty had reduced deep vein thrombosis by an average of 80% compared with conventional hip replacements.^24^

### Current evidence

The published literature, however, suggests that the rate of deep vein thrombosis is similar for resurfacing arthroplasty and hip replacements.^21,34^

### ‘New hip let me have a baby’

In August 2002 the *Daily Mirror* claimed that hip resurfacing arthroplasty makes the hips flexible enough to allow natural delivery without any difficulties.^18,26^

### Current evidence

A restricted range of movement after hip resurfacing arthroplasty and the factors causing it have been well documented in in vitro^41^ and clinical studies.^42^ It has been shown that cobalt and chromium are able to cross the placenta in patients with a metal-on-metal hip resurfacing arthroplasty although the placenta does have a modulatory effect on the rate of transfer.^11^ In a study on the effect of metal ions on reproduction and development, it was shown that the action of chromium ions may in fact be relevant in several stages of pregnancy, leading to subfertility, infertility, intrauterine growth retardation, spontaneous abortions, malformations, birth defects, postnatal death, learning and behaviour deficits, and premature ageing.^43^ In contrast, normal vaginal delivery is feasible and safe after a conventional total hip replacement.^44^

### ‘High failure rate’

In September 2008 *The Times* and the *Daily Mail* raised safety concerns about hip resurfacing, claiming that the ‘quick-fit’ hip resurfacing arthroplasties were failing in three years.^45,46^ Subsequent reports have highlighted the high failure rate with hip resurfacing arthroplasty.^47–52^ Hip resurfacing arthroplasty has been quoted as having ‘double the expected failure rate’^52,53^ with some clearly identifying the ‘lower success rate than older, more established methods, which replace the entire joint’.^46^

### Current evidence

The eighth annual report of the NJR revealed that the five-year revision rate for hip resurfacing arthroplasty was 6.3% compared with 2% for cemented hip replacements.^6^

### ‘High metal ions’

In November 2009 *The Times* reported the side effects of chromium and cobalt ion release with hip resurfacing arthroplasty and that there is a concern that ‘the leaching of these metals into the rest of the body may prove harmless, but there are suspicions that, as well as causing localised tissue and bone damage, it could have a detrimental effect on other organs in the body’.^47^

### Current evidence

Raised metal ion levels after hip resurfacing arthroplasty are well documented.^54–56^ These tend to stabilise three^55^ to six months^54^ after surgery. Research has shown that metal ion concentrations that are not directly cytotoxic to lymphocytes may affect events at a molecular level, thereby impeding lymphocyte proliferation.^56^ This may contribute to altered immune system function in patients with cobalt-chromium implants.^56^

### ‘Tumour fear’

In March 2010 the *Daily Mail* reported that ‘hip resurfacing arthroplasty can wreak havoc with your body’ and may leave patients ‘crippled for life’.^57^ It was also feared that the implant could cause tumours and tissue damage.^49–51^ Mention of aseptic lymphocytic vascular and associated lesions (ALVAL) and inflammatory pseudotumours have been made.^57^

### Current evidence

The risk of pseudotumours has been reported to range from 0.15% to 1.8% after metal-on-metal hip resurfacing arthroplasty.^12,58^ In a multi-surgeon series of 5,000 hip resurfacing arthroplasties, Carrothers *et al* reported the incidence of revision for ALVAL/metallosis to be about 0.3%, with women being at a significantly higher risk (*p*=0.01).^39^

## Discussion

This study shows that there is disparity between the claims in the newspapers and the published scientific literature. The initial newspaper articles highlighted only the positive aspects of hip resurfacing arthroplasty without definitive evidence backing the claims. The reports from 2008 onwards concentrated on the high revision rate, metal ion issues and fears of tumour.^45–53,57,59^ During the same period, the eighth annual report of the NJR confirmed that the trend for the use of hip resurfacing arthroplasty reversed (Fig 1).^6^ It is not clear if the trend changed because of the decrease in surgeon preference raised by concerns in the scientific literature or a reduction in patient demand created by the negative publicity in the popular press apparent from mid-2008. The media reports would clearly impact the practice of surgeons too but the reversal in trend of the number of resurfacings implanted began before the media publicity.

One would expect the effect of newspaper reports to appear in the period somewhat after the publication and not the very same year. The patients who are in need of hip surgery would be the ones reading the newspaper articles and from the time of referral by their general practitioner (followed by attendance at an orthopaedic clinic and a decision being made about surgery) to the date of the actual operation, there would be a lag from the time of publication of the article.

The increasing trend of hip resurfacing coincides with the positive media reports in the previous years. Fewer hip resurfacing arthroplasties were performed in 2008 than in 2007,^6^ suggesting that the decreasing trend actually predates the adverse newspaper reports. The surgeons would be in the privileged position to react immediately to the adverse scientific evidence and take appropriate measures. It can therefore be extrapolated that the numbers reduced because of publications in the scientific literature rather than the newspapers. The trend may also reflect a delay in acceptance of positive reports and an immediate response to negative reports.

We have also found that the media publications in the early years contradict the information reported later. Several claims were made that were clearly exaggerated and not backed by contemporaneous literature. The hopes that hip resurfacing arthroplasty is an everlasting solution, less invasive, less time consuming or allows women to have babies have been contradicted. A systematic review in 2011 even questioned whether hip resurfacing arthroplasty is better than conventional hip replacements for young active patients.^32^ In recent years, the newspapers have reported information that can be supported by the scientific literature, of which the surgeons were already aware but to which the media reacted slowly. The media have helped spread this crucial information but not highlighted the success of certain prostheses that have stood the test of time.

The study is limited by a lack of demographic/normative impact analysis. The raw circulation figures were used, which, however blunt, are probably the quickest and easiest markers available. The circulation figures do not necessarily equate to the number of readers as it may be that several people read the same newspaper. Moreover, the readership of the newspapers varies and whether someone reads an article or not depends on individual interest and a possible need for hip surgery. The impact of the page on which an article is published is also a factor to consider. The direct influence of the media reports on the clinicians may also be a factor to consider.

The power of the media should be directed towards improving the spread of scientific knowledge in order to encourage behavioural changes.^3^ The media and the internet are powerful means of spreading medical information; this should come with greater accountability. Strategic efforts are needed to improve the quality of medical news reporting by the media and to provide guidance for patients to understand their disease and interpret such information better.^60^

The scientific community also has a responsibility in using the media in a constructive way in order to disseminate information. While it is imperative to announce significant findings, it is equally crucial that the general public is able to weigh and interpret these appropriately. Patients who obtain scientific information from newspapers must be aware that the decision making process needs to account for individual circumstances. Clinicians are better placed to judge the quality of research and the application of this in their practices.

## Conclusions

The trends of the newspaper articles and of the number of hip resurfacing arthroplasties implanted suggest that the media may have been partly responsible for the drive to increase the use of this prosthesis. The subsequent decrease was initiated by the scientific literature.
